# Hybrid Mesoporous Silicas and Microporous POSS-Based Frameworks Incorporating Evaporation-Induced Self-Assembly

**DOI:** 10.3390/nano5021087

**Published:** 2015-06-16

**Authors:** Jheng-Guang Li, Wei-Cheng Chu, Shiao-Wei Kuo

**Affiliations:** Department of Materials and Optoelectronic Science, Center for Nanoscience and Nanotechnology, National Sun Yat-Sen University, Kaohsiung 804, Taiwan; E-Mails: d983100007@student.nsysu.edu.tw (J.-G.L.); m993100044@student.nsysu.edu.tw (W.-C.C.)

**Keywords:** mesoporous, polyhedral oligomeric silsesquioxane (POSS), evaporation-induced self-assembly

## Abstract

We fabricated a series of mesoporous silicas and mesoporous organosilicates with hierarchical porosity through evaporation-induced self-assembly using Pluronic F127 as a template in this study. We could tailor the mesophase of each mesoporous silica sample by varying the weight ratio of its two silica sources: tetraethyl orthosilicate (TEOS) and triethoxysilane hydrosilylated octavinyl polyhedral oligomeric silsesquioxane (OV-POSS-SILY). The mesophases ranged from an ordered body-centered cubic (*bcc*) structure (TEOS alone) to ordered face-centered cubic (*fcc*) structure (10 and 20 wt.% of OV-POSS-SILY) and finally to disordered spherical pores (≥30 wt.% of OV-POSS-SILY). We used small-angle X-ray scattering (SAXS) and transmission electron microscopy (TEM) to study the transformations of these mesophases, while N_2_ isotherm sorption curves revealed the porosities of these mesoporous silicate samples. Moreover, ^29^Si CP/MAS solid state nuclear magnetic resonance spectroscopy allowed us to analyze the compositions of the POSS-containing silicate frameworks. Such functional mesoporous silica samples incorporating microporous POSS building units have potential applications in various systems, including optical and electronic devices.

## 1. Introduction

**Scheme 1:**
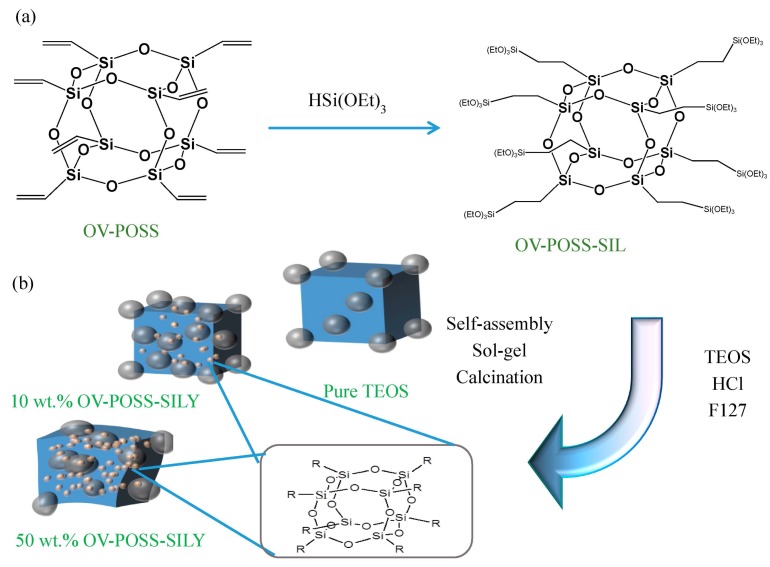
(**a**) Synthesis of triethoxysilane hydrosilylated octavinyl polyhedral oligomeric silsesquioxane (OV-POSS-SILY); and (**b**) The preparation of mesoporous silicas with different amounts of polyhedral oligomeric silsesquioxane (POSS) building units tempalted by F127 triblock copolymers.

Polyhedral oligomeric silsesquioxane (POSS) has received much attention recently because of its unique cage-like structure (intramolecular pore size: *ca.* 0.3 nm) and its interesting phase behavior and properties at multiple length scales [1,2,3,4,5,6,7,8,9,10,11,12,13,14]. This well-defined nanoscale organic/inorganic structure is an ideal building block for fabricating nanostructured hybrid materials and nanocomposites [14,15]. For example, many hierarchically porous materials have been developed to take advantage of the characteristics of POSS, with various applications envisaged in the fields of catalysis [16], adsorption [17], photonics [18], and electronics [19,20]. There are several examples of using POSS-based frameworks in the fabrication of novel mesoporous silica materials. Zhang *et al.* co-assembled triethoxysilane hydrosilylated octavinyl polyhedral oligomeric silsesquioxane (OV-POSS-SILY; Scheme 1) with poly(ethylene oxide)-*b*-(propylene oxide)-*b*-(ethylene oxide) (PEO_20_PPO_70_PEO_20_, P123) micelles to fabricate a mesoporous organic/inorganic hybrid material having a hierarchical architecture and various functionality (including the cubic silsesquioxane cage, the bridging ethylene groups, and the pendant vinyl groups) [21]. Furthermore, they performed more detailed characterization and examined the deeper applications of a bifunctional super-microporous organosilica prepared through the assembly of predefined POSS nano building blocks around a poly(ethylene glycol) octadecyl ether (Brij-76) template [22]. This super-microporous structure arose from the specific shape and hydrophobic characteristics of the POSS nanoparticle, moreover, the reactive vinyl groups of octavinyl POSS that had not undergone hydrosilylation remained highly accessible and reacted readily with Br_2_. More recently, using an evaporation-induced self-assembly (EISA) spin-coating procedure, the Ozin group reported a new periodic mesoporous organosilica (PMO) derived from POSS that they prepared through self-assembly between the surfactant cetyltrimethylammonium chloride (CTACl) and the PMO precursor [23]. Here, hierarchical porosity was induced by the pores (*ca.* 1.5 nm) that appeared after removal of CTACl and by the cubic-cage structures within the pore walls, thereby lowering the dielectric constant (*k*) and the refractive index.

The EISA strategy has been developed aggressively in recent years [24,25,26,27,28]. Its combination of templated mesophase growth and evaporation of volatile solvents can extend the scope of available mesoporous materials through variations of the types of material frameworks and structure-directing agents. In previous studies, we applied the EISA approach with various templates and matrices; the use of volatile organic solvents—in most cases tetrahydrofuran (THF)—allowed the application of templates having high molecular weight, including PEO-*b*-PCL, PEO-*b*-PLLA, PE-*b*-PEO, PE-*b*-PEO-*b*-PCL, and F127 (PEO_106_PPO_70_PEO_106_) [29,30,31,32,33,34,35,36]. In addition to traditional silica materials, mesoporous organic materials also can be fabricated using the EISA method, including mesoporous phenolic resin [37,38,39,40,41], mesoporous polybenzoxazine [42], and mesoporous carbon [43,44,45,46].

Previously, we used the EISA method to obtain an ordered body-centered cubic (*bcc*) mesoporous silica that had been templated by Pluronic F127 [35]. Furthermore; we had also employed PEO-*b*-PCL as a template and incorporated a star-shaped PEO-POSS as a mesophase transfer agent during the synthesis of a mesoporous phenolic resin. In this present study, we synthesized the organosilica precursor OV-POSS-SILY through hydrosilylation of OV-POSS [20,21,22]. POSS-based porous organosilicas ordinarily exhibit short-range order, possibly because of poor mobility or compatibility during the co-assembly process with templates [6,7,8]. Here, we used F127 as the template and regulated the mesophase of mesoporous silica through the application of two different silica sources: tetraethyl orthosilicate (TEOS) and OV-POSS-SILY. We maintained the weight ratios of the silica precursor to F127 at 3:1 and varied the weight ratios of OV-POSS-SILY to TEOS (0, 10, 20, 30, 50, or 100 wt.%, as shown in Scheme 1). We observed the clear mesophase transformation upon varying the weight ratios of OV-POSS-SILY: the nanostructure has a *bcc* form when prepared using TEOS alone, becomes face-centered cubic (*fcc*) when using 20 wt.% OV-POSS-SILY, and eventually formed a disordered spherical structure when using 100 wt.% OV-POSS-SILY. We employed ^1^H, ^13^C, and ^29^Si nuclear magnetic resonance (NMR) spectroscopy to monitor the synthesis of the silica precursor (OV-POSS-SILY); small-angle X-ray scattering (SAXS) and transmission electron microscopy (TEM) to analyze the mesophase of the mesoporous silicas; N_2_ sorption isotherms to study the porosity of the mesoporous materials; and ^29^Si cross polarization/magic angle spinning (CP/MAS) solid state NMR spectroscopy to determine the compositions of the silica frameworks. These analyses indicate that mesoporous thin films having hierarchical porosities and reactive vinyl groups might be useful materials in a variety of fields.

## 2. Results and Discussion

**Figure 1:**
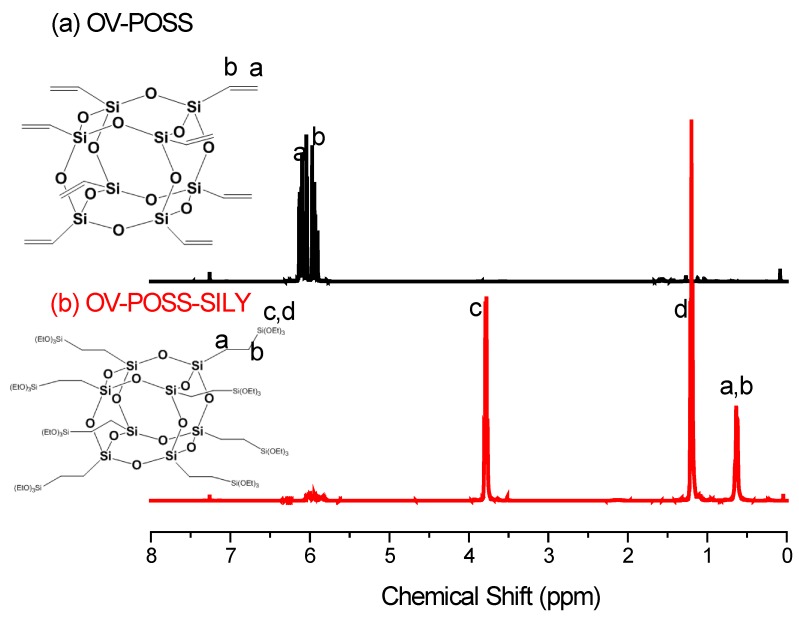
^1^H nuclear magnetic resonance (NMR) spectra of OV-POSS and OV-POSS-SILY.

**Figure 2:**
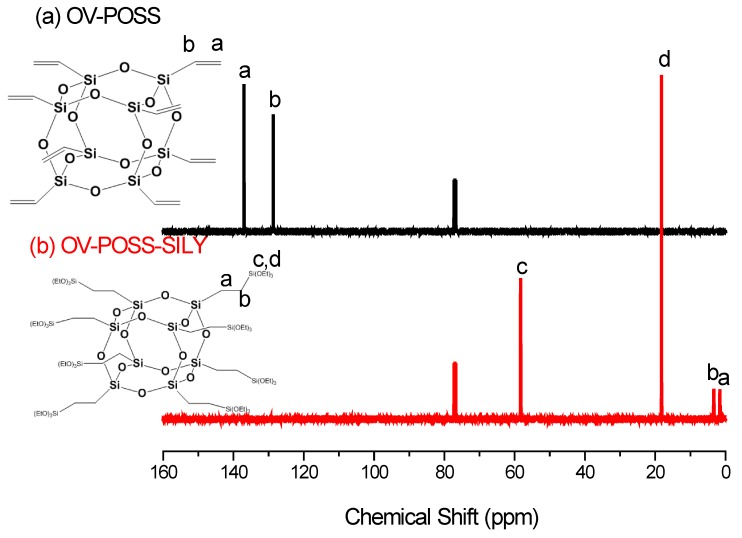
^13^C NMR spectra of OV-POSS and OV-POSS-SILY.

**Figure 3:**
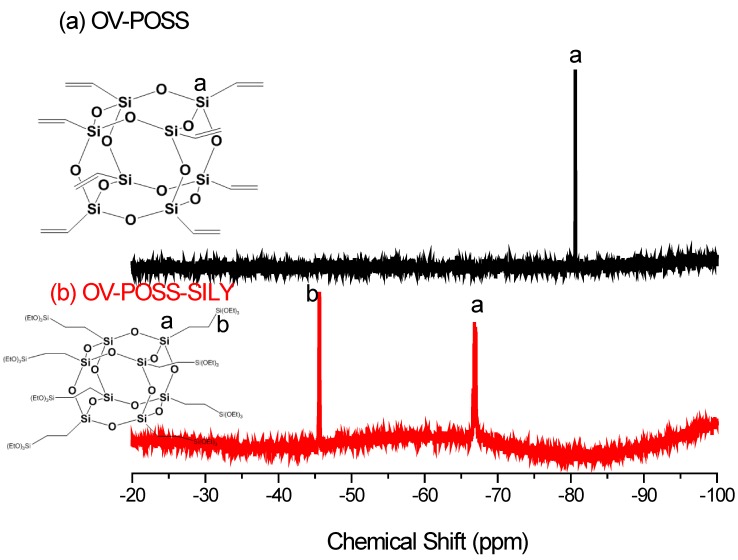
^29^Si NMR spectra of OVPOSS and OV-POSS-SILY.

We firstly synthesized OV-POSS-SILY from OV-POSS, a commercial octavinyl-substituted POSS octamer. Figure 1, Figure 2 and Figure 3 present the ^1^H, ^13^C, and ^29^Si NMR spectra, respectively, of OV-POSS and OV-POSS-SILY; in each case, the signals of OV-POSS disappeared almost completely after hydrosilylation. As revealed in Figure 1, only a small number of vinyl groups (between 5.8 and 6.0 ppm) remained, implying that almost all of the vinyl groups of OV-POSS had reacted to form OV-POSS-SILY. A simple calculation of the integral areas of the ethoxy and vinyl groups revealed that the degree of hydrosilylation was 93.6%.

In the ^13^C NMR spectra (Figure 2), the signals for the vinyl groups of OV-POSS at 128.7 and 136.9 ppm also almost disappeared completely, with the appearance of signals for the corresponding CH_2_ and CH_3_ groups of OV-POSS-SILY at 1.69, 3.39, 18.2, and 58.2 ppm. In the ^29^Si NMR spectra (Figure 3), the peak at −80 ppm for OV-POSS disappeared completely, with new signals appearing for OV-POSS-SILY at −66.8 and −45.4 ppm. Thus, the ^1^H, ^13^C, and ^29^Si NMR spectra collectively confirmed the successful synthesis of OV-POSS-SILY.

**Figure 4:**
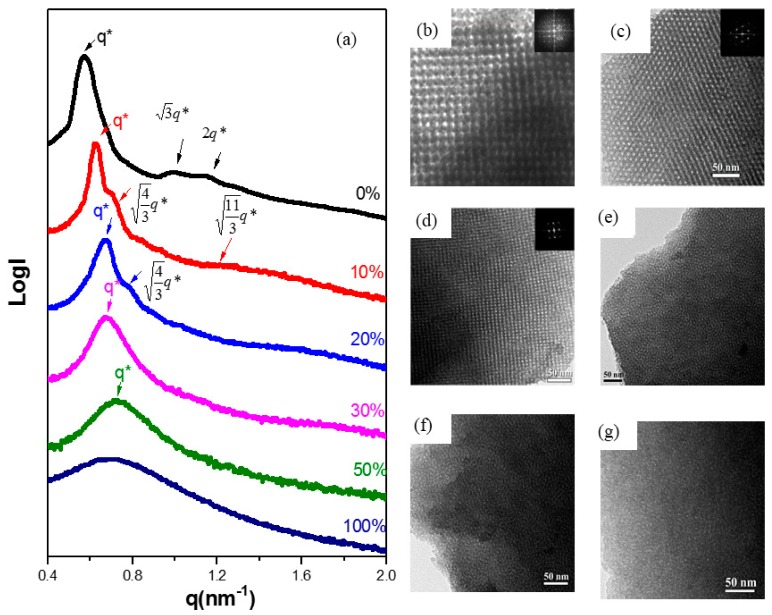
(**a**) Small-angle X-ray scattering (SAXS) and transmission electron microscopy (TEM) images of mesoporous silica samples templated by F127 at various OV-POSS-SILY-to-silica precursor weight ratios of (**b**) 0, (**c**) 10, (**d**) 20, (**e**) 30, (**f**) 50, and (**g**) 100 wt.%.

Previously, we used an efficient EISA method to prepare ordered *bcc* mesoporous silica when using F127 as the template and TEOS as the silica source (Figure 4b), 0 wt.% of OVPOSS-SILY) at a TEOS-to-F127 weight ratio of 3:1.10 (g). For this present study, we incorporated OV-POSS-SILY as a novel silica source to prepare a series of mesoporous silicas, having different mesophases and silica frameworks, when using 0, 10, 20, 30, 50, and 100 wt.% OV-POSS-SILY at a constant silica source-to-F127 weight ratio of 3:1.

Figure 4 presents SAXS patterns and TEM images, respectively, of the mesoporous silicas prepared with various OV-POSS-SILY weight ratios in the silica source. Two features are evident in the SAXS patterns (Figure 4a). First, the character of the reflection ratio transformed completely upon varying the OV-POSS-SILY weight ratio, indicating that the mesophase could be changed by regulating the amount of OV-POSS-SILY. Second, the *d*-spacing, calculated from the primary scattering peak, shifted accordingly, suggesting a decrease in the size of the unit cell. Prior to the addition of OV-POSS-SILY, the original mesoporous silica had a typical *bcc* structure, with a scattering ratio of 1:3^1/2^:2. A dramatic variation in the structure appeared when using 10 wt.% of OV-POSS-SILY in the silica source: the scattering ratio moved from 1:3^1/2^:2 to 1:(4/3)^1/2^:(11/3)^1/2^ with the appearance of the peak (4/3)^1/2^ strongly suggesting that the mesophase had transformed from the *bcc* structure to the *fcc* structure. The SAXS pattern of mesoporous silica prepared in the absence of OV-POSS-SILY revealed a *bcc* structure; the addition of OV-POSS-SILY at 10 or 20 wt.% caused a transformation into an *fcc* structure, which became a short-range-ordered disordered sphere structure upon increasing the OV-POSS-SILY weight ratio in the silica precursor to 30, 50, or 100 wt.%. In other words, the order of the mesoporous silica could be destroyed when adding an excessive quantity of OV-POSS-SILY. On the other hand, the primary scattering peak of the mesoporous silica samples tended toward larger values of *q* upon increasing the amount of OV-POSS-SILY in the silica precursor, with the *d*-spacing of the mesopore arrangement decreasing from 10.8 to 8.66 nm (Table 1). The presence of the OV-POSS-SILY units, which are somewhat star-shaped, shrank the unit cell of the mesostructure. TEM images of the mesoporous silica samples were perfectly consistent with the SAXS patterns. The sample prepared using TEOS alone as the silica precursor featured the (100) phases of a typical *bcc* structure (Figure 4b). After increasing the amount of OV-POSS-SILY to 10 or 20 wt.%, the TEM images revealed the character of an *fcc* structure (Figure 4c,d).

**Table 1:** Textural properties of mesoporous silicas templated by F127 at various OV-POSS-SILY weight ratios.

OV-POSS-SILY	*d* (nm)	Pore Size (nm)	*S*_BET_ (m^2^/g)	*S*_M_ (m^2^/g)	Pore Volume (cm^3^/g)	Micropore Volume (cm^3^/g)
0%	10.8	4.8	831	185	0.71	0.076
10%	9.97	4.0	809	264	0.49	0.114
20%	9.35	3.4	622	272	0.34	0.121
30%	9.35	2.5	418	243	0.21	0.112
50%	8.66	-	537	313	0.32	0.145
100%	-	-	519	252	0.26	0.115

*d*-spacings were calculated using *d* = 2π/q*; *S*_BET_ and *S*_M_ are the total Brunauer, Emmett and Teller (BET) surface area and micropore surface area, respectively.

Unfortunately, the structure became disordered when we increased the OV-POSS-SILY weight ratio further, to 30 wt.% (Figure 4d). Thus, from the SAXS patterns and TEM images, we conclude that an increase in the content of OV-POSS-SILY expanded the volume fraction of the outer domain efficiently; the resulting structural transformation from *bcc* to *fcc* implies that the presence of OV-POSS-SILY induced closer packing of the F127 micelles.

**Figure 5:**
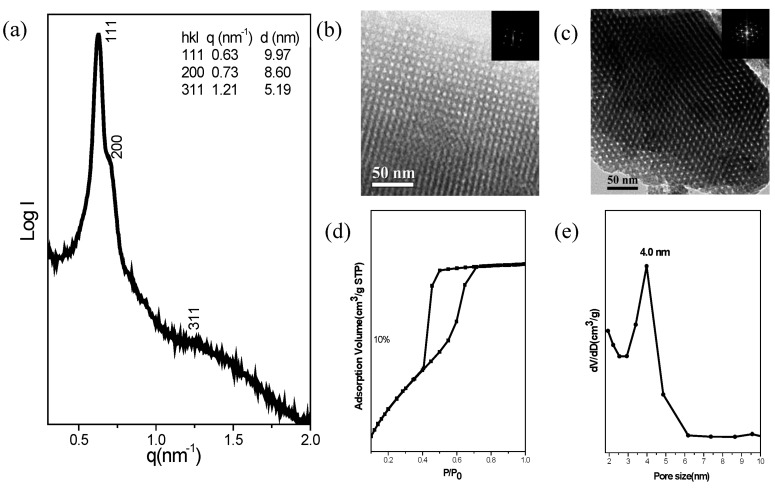
(**a**) SAXS; (**b**) TEM image viewed from (100); (**c**) TEM image viewed from (110) (insets: corresponding FFT); (**d**) N_2_ adsorption/desorption isotherm; and (**e**) Pore size distribution curve of the *fcc* mesoporous silica templated by F127 at a TEOS/OV-POSS-SILY (10 wt.%)-to-F127 ratio of 3:1.

Figure 5 presents characterization data for the *fcc* mesoporous silica sample prepared using 10 wt.% of OVPOSS-SILY. The SAXS pattern (Figure 5a) of this *fcc* mesoporous silica displays a strong reflection with the large *d*-spacing of 9.97 nm, combination of two reflections at *q* = 0.63 nm^−1^, and 0.73 nm^−1^; could be indexed as having (111), (200), and (311) reflections, corresponding to a cubic structure (Fm$\bar{3}$m space group). In addition, we also use the TEM analysis (Figure 5b,c) to determine the structure ordering and cubic symmetry of this mesoporous silica. This *fcc*-type mesoporous silica with different orientations ((100) and (110) planes) is consistent with a three-dimensional (3D) cubic cage structure. Figure 5d shows the N_2_ adsorption/desorption isotherm measured at 77 K to obtain the further information about the textural properties of this cubic mesoporous silica. We observe individual type-IV isotherms with an apparent H_2_ hysteresis loop characteristic of a cage-like mesoporous material. The sharp capillary condensation step was appeared at a relative pressure (*P*/*P*_0_) of approximately 0.65, indicating the uniform pore dimensions and high-quality ordering of the materials. Pore size distribution analysis shows a well-ordered cubic structure having pores with an average diameter *ca.* 4.0 nm (Figure 5e). Using the Broekhoff–de Boer (BdB) model to calculate the sizes of its spherical pores, the BET surface area of the sample was 809 m^2^/g; the pore volume was approximately 0.49 cm^3^/g (Table 1, 10 wt.%).

**Figure 6:**
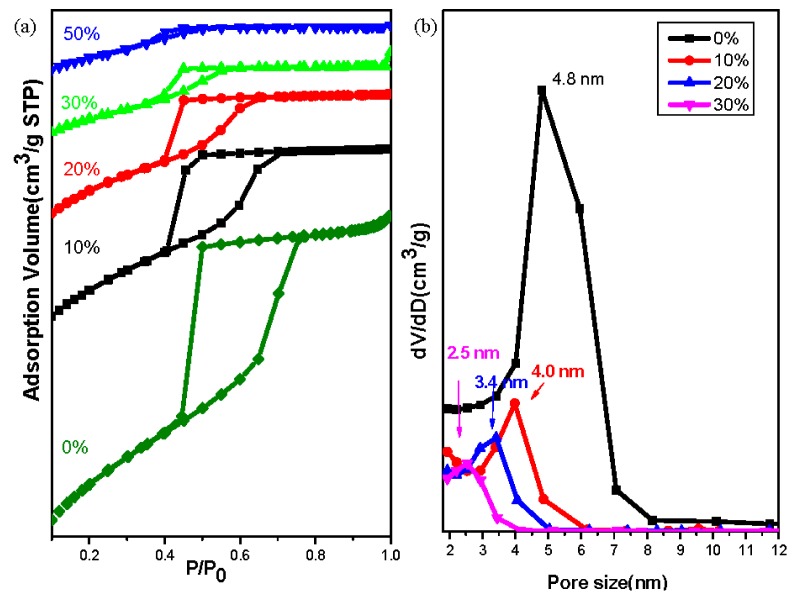
(**a**) N_2_ adsorption/desorption isotherms; and (**b**) Pore size distribution curves of mesoporous silica samples templated by F127 at various OV-POSS-SILY weight ratios.

The N_2_ sorption isotherms of mesoporous silica samples obtained at the various silica precursor ratios exhibited (Figure 6) representative type-IV curves with sharp capillary condensation steps in the relative pressure range from 0.60 to 0.75, especially for the samples prepared in the presence of 0, 10, and 20 wt.% of OV-POSS-SILY (with *bcc*, *fcc*, and *fcc* structures, respectively), implying uniform distributions of their pore sizes. The hysteresis loops of these mesoporous silica samples tended to shrink, however, upon increasing the ratio of OV-POSS-SILY, suggesting that the porosity decreased accordingly, ultimately resulting in the pore size distribution being barely measurable when the OV-POSS-SILY weight ratio extended beyond 30 wt.% (Figure 6b). Combining the results from the N_2_ sorption isotherms and the TEM images, we found that the mesopores of the mesoporous silica samples prepared using 50 and 100 wt.% of OV-POSS-SILY were mostly close pores, due to the behavior of the hysteresis loop, suggesting that the F127 micelles had assembled more densely and that the polymer chains were confined within the silicas. The pore size distribution curve, measured from the adsorption branches based on the BdB model, is consistent with this explanation. Clearly, the pore size varied from 4.8 to 2.5 nm upon increasing the OV-POSS-SILY ratio. Table 1 summarizes the textural properties of our mesoporous silica samples; the trends in the *d*-spacing and pore sizes correlated with the shrinkage of the unit cell of silica samples upon increasing the OV-POSS-SILY weight ratio. The BET surface area decreased from 801 m^2^/g for the sample prepared using TEOS alone to 418 m^2^/g for the sample prepared using 30 wt.% OV-POSS-SILY, increased slightly to 537 m^2^/g for the sample prepared at 50 wt.%, and eventually stabilized at 519 m^2^/g for the sample prepared using OV-POSS-SILY alone. In contrast, the micropore surface area grew continuously (from 185 to 313 m^2^/g) upon increasing the OV-POSS-SILY ratio, indicating that the POSS components in the silica walls were certainly contributing to the content of micropores. In addition, the collapse of the mesophase might have been the reason for the decrease in the total surface area. Furthermore, the decreases in both the total surface area and micropore surface area are consistent with not only the poorer structure of the mesophase but also the destruction of the silica framework during the calcination step.

**Figure 7:**
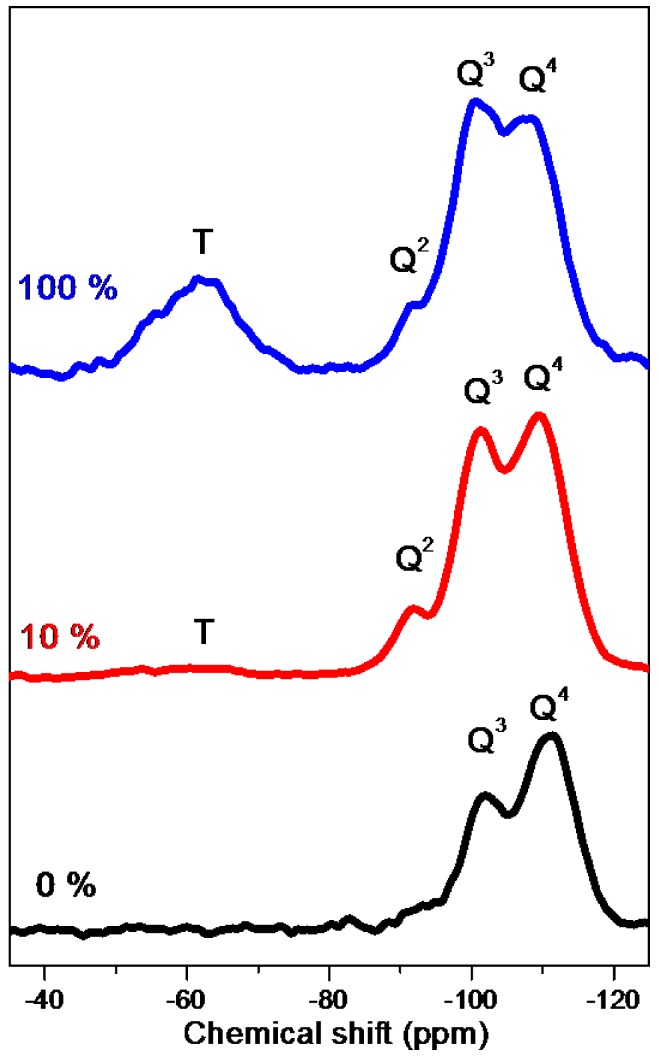
^29^Si CP/MAS solid state NMR spectra of mesoporous silica samples prepared using 0, 10, and 100 wt.% of OV-POSS-SILY.

To determine the composition of the silica framework, we used 29Si CP/MAS solid state NMR spectroscopy to analyze the samples prepared using 0, 10, and 100 wt.% of OV-POSS-SILY (Figure 7). For the sample prepared using OV-POSS-SILY alone, we attribute the broad *T_n_* signals to a blend of CSi(OSi)_3_ (*T*_3_), CSi(OSi)_2_O*R* (*T*_2_), and CSi(OSi)(O*R*)_2_ (*T*_1_) units, where *R* is either H or Et. The *T* peak reveals that, to some extent, the cubic cage structure of POSS remained. The ^29^Si solid state NMR spectrum also features three *Q_n_* signals at −111, −102, and −92 ppm, which we assign to Si(OSi)_4_ (*Q*_4_), Si(OSi)_3_O*R* (*Q*_3_), and Si(OSi)_2_O*R*_2_ (*Q*_2_) units, indicating that Si–C cleavage occurred to a certain degree during either the hydrothermal step or the calcination process [7]. The *T*/*Q* ratio of the sample prepared using OV-POSS-SILY alone, calculated from the integral area fractions of the *T_n_* and *Q_n_* signals, was 0.25, suggesting that 25% of the cage-like structures remained after sequential treatment through EISA and hydrothermal and calcination processes. In contrast, the *T_n_* intensity ratio for the sample prepared using 10 wt.% of OV-POSS-SILY was much smaller; its calculated ratio of integral areas of *T* and *Q* signals was 0.025, meaning that the cage-POSS structures comprised only 2.5% of the silica framework. For the sample prepared using TEOS alone, the disappearance of the *T_n_* signals is consistent with the absence of OV-POSS-SILY from the feed ratio; in other words, the absence of OV-POSS-SILY resulted in the presence of only pure *Q_n_* signals.

## 3. Experimental Section

### 3.1. Materials

OV-POSS was purchased from Hybrid Plastics. Triethoxysilane [HSi(OEt)_3_, 95%], platinum(0)-1,3-divinyl-1,1,3,3-tetramethyldisiloxane [Pt(dvs)] complex solution in xylenes (*ca.* 2% Pt), and Pluronic F127 (EO_106_PO_70_EO_106_; molecular weight: 12,600) were purchased from Aldrich. Toluene was distilled over sodium prior to use. TEOS (99%) and hydrochloric acid were obtained from SHOWA. EtOH (95%) and THF (>99%) were purchased form ECHO.

### 3.2. OV-POSS-SILY

Triethoxysilane (3.60 mL, 5.19 g, 31.6 mmol) and Pt(dvs) complex solution in xylene (10 μL) were added sequentially to a solution of OV-POSS (2.00 g, 3.16 mmol) in toluene (20 mL) and then the mixture was stirred at 80 °C for 12 h. After cooling to ambient temperature, the solvent and the unreacted triethoxysilane were evaporated under vacuum. The resulting hydrosilylated product was a colorless and sticky oil. ^1^H NMR (500 MHz, CDCl_3_): δ 0.6 (t, 28H, ethylene), 1.2 (t, 63H, CH3 of ethoxy), 3.8 (q, 42H, CH2 of ethoxy), 6.0–5.8 (m, 9H, vinyl). ^13^C NMR (500 MHz, CDCl_3_): δ 1.6, 3.4, 18.2, 58.3. ^29^Si NMR (500 MHz, CDCl_3_): δ −45.6, −66.8.

### 3.3. Mesoporous Silicas Containing POSS

Syntheses were performed at various ratios of OV-POSS-SILY to TEOS (0, 10, 20, 30, 50, and 100 wt.%), a constant silica precursor-to-F127 weight ratio (3:1) at a constant HCl(aq) concentration. The silica precursor (a mixture of OV-POSS-SILY and TEOS) was added to a solution of the block copolymer in THF with stirring for 30 min. The sample was poured into a Petri dish and the THF solvent was evaporated over 48 h at room temperature. The transparent film was collected and ground into a powder, which was transferred to a Perfluoroalkoxy alkanes (PFA) bottle containing 1.0 M HCl and treated hydrothermally at 100 °C for one day. The product was washed with water and ethanol, dried at room temperature, and calcined in air at 500 °C for 1 h to produce mesoporous silica material. Calcination processes were performed in a furnace at the heating rate of 1 °C/min.

### 3.4. Characterization

^1^H, ^13^C, and ^29^Si solution NMR spectra were used the Bruker AM 500 (500 MHz) spectrometer (Bruker Corporation, Billerica, MA, USA) and the deuterated solvent acting as the internal standard. ^29^Si MAS solid state NMR spectra were used the AVIII 600 WB solid state NMR spectrometer (Bruker Corporation, Billerica,MA, USA). SAXS experiments were located at the BL17B3 beamline of the NSRRC, Taiwan. The X-ray beam had a diameter of 0.5 mm and a wavelength (λ) of 1.24 Å. TEM samples were suspended in EtOH and supported onto a holey carbon film on a Cu grid by using a JEOL 3010 microscope (JEOL Ltd., Tokyo, Japan) operated at 200 kV. Nitrogen adsorption/desorption isotherms were determined at −196 °C using an ASAP 2020 analyzer (Micromeritics Instrument Corporation, Norcross, GA, USA). The samples were degassed under vacuum at 200 °C for at least 6 h prior to measurements. The specific surface areas and pore volumes; pore size distributions were derived from the adsorption branches of the isotherms by using the Barrett-Joyner-Halenda (BJH) model.

## 4. Conclusions

We present herein the first example of the construction of POSS-based mesoporous silica samples with specific mesophase structures that could be changed upon regulation of the OV-POSS-SILY weight ratio in the silicate precursor when using a simple EISA method. Although destruction occurred during the thermal treatment process, some degree of the POSS structures remained, as evidenced through characterization using ^29^Si CP/MAS solid state NMR spectroscopy. Because of the bifunctional characteristics, the porosity enhancement induced by the cage-like POSS within the silica walls, and the remaining vinyl groups from the OV-POSS-SILY units, these mesoporous organosilicas having ordered structures might have potential applications as low-*k* interlayer dielectric materials or in catalysis.
